# Treatment of Haemophilus influenzae Serotype A (Hia) Meningitis in a Pediatric Patient: A Case Report

**DOI:** 10.7759/cureus.80253

**Published:** 2025-03-08

**Authors:** Matthew Thomas, Shivang Patel, Harthik Kambhampati, Miriam Thomas

**Affiliations:** 1 Pediatrics and Child Health, Lake Erie College of Osteopathic Medicine, Bradenton, USA; 2 College of Medicine, Lake Erie College of Osteopathic Medicine, Bradenton, USA; 3 Pediatrics, Saint Clare’s Denville Hospital, Denville, USA

**Keywords:** cadvt, empiric antibiotics, haemophilus meningitis, meningitis seizure, meningitis siezure, pediatric infectious disease

## Abstract

*Haemophilus influenzae* serotype A (Hia) meningitis is a rare but severe pediatric infection, with significant risks of mortality and long-term complications. Here, we describe a 10-week-old male presenting with Hia meningitis complicated by cerebritis, seizures, and central venous catheter-related deep vein thrombosis (CADVT). The patient initially presented with fever, irritability, and feeding difficulties and was later found to have significant laboratory abnormalities and cerebrospinal fluid (CSF) findings suggestive of bacterial meningitis. This case highlights the empiric treatment of pediatric meningitis, tailored and adjunctive treatments based on culture results, and other thrombotic and neurological complications. Despite high morbidity risks, the patient demonstrated complete clinical recovery at two years and showed no signs of long-term sequelae. This case emphasizes the evolving epidemiology of Hia and the importance of timely diagnosis and multidisciplinary management in severe pediatric infections, to ensure the minimization of negative externalities.

## Introduction

Bacterial meningitis develops when a pathogen invades the subarachnoid space, causing inflammation of the meninges, particularly the pia mater and arachnoid mater. It is a devastating illness, especially in neonates and infants. Bacterial meningitis has a high case-fatality rate of up to 30%, and as many as 50% of survivors develop neurological complications, with outcomes highly dependent on the patient’s age and the infecting organism [1]. The primary mode of transmission for *Haemophilus influenzae (H. influenzae)* is through the inhalation of respiratory droplets from an infected individual or by direct close contact. From there, *H. influenzae* can enter the bloodstream, leading to bacteremia. *H. influenzae* exists in two distinct forms: encapsulated strains (serotypes a-f), which possess protective polysaccharide capsules that enable the bacteria to evade phagocytosis and circulate in the bloodstream, and non-encapsulated, nontypeable strains (NTHi), each of which contributes uniquely to disease patterns and severity [2].

For the past 20 years, encapsulated serotypes have been more frequently identified as contributors to severe invasive infections, including Hia [1]. Historically, *Haemophilus influenzae* serotype b (Hib) was the leading cause of bacterial meningitis and a major factor in pneumonia among children under five [2]. However, in the 1980s, the introduction of the polyribosyl ribitol phosphate (PRP) protein-conjugate Hib vaccine caused a major decline in Hib infections. Despite the development of the PRP protein-conjugate Hib vaccine, sporadic outbreaks are still seen in various countries, especially those with lower vaccine coverage.

Here, we highlight the management of a 10-week-old child with Hia meningitis, complicated by cerebritis and CADVT. This case is significant due to the rarity and severity of Hia meningitis, particularly with this case's complications, which highlights the importance of timely, tailored treatment and multidisciplinary management.

## Case presentation

We present a 10-week-old male who arrived at his pediatrician’s office with a history of decreased feeding, irritability, and fever. Earlier that day, his mother noticed behavioral changes, including reduced milk intake (4 oz compared to his usual 20 oz) and increased crankiness. A rectal temperature of 101 °F was recorded at home. The patient first visited his pediatrician and was given Tylenol, though no definite source of infection was identified. Due to the persistence of symptoms without an identifying cause, the pediatrician referred the patient to the emergency department for further evaluation.

Upon arrival at the emergency department, several tests were run on the patient. First, rapid antigen testing for Influenza A/B and respiratory syncytial virus (RSV) was negative. Further laboratory testing revealed several significant abnormalities. A complete blood count (CBC) revealed leukopenia (white blood cells 1.60 × 10³/µL), mild anemia (hemoglobin 9.4 g/dL, hematocrit 26.8%), and a moderate elevation in red cell distribution width (13.5%) (Table 1). A comprehensive metabolic panel was also collected, revealing hyponatremia (Na 132 mmol/L), hyperglycemia (glucose 185 mg/dL), and a blood urea nitrogen (BUN)/creatinine ratio of 43.3 (Table 2). Liver function tests (LFTs) revealed low serum protein (total protein 5.6 g/dL), hypoalbuminemia (3.1 g/dL), as well as slightly elevated liver enzymes (alanine transaminase (ALT) 22 U/L, aspartate aminotransferase (AST) 47 U/L) (Table 3). A urinalysis was performed, revealing cloudy fluid with the presence of trace ketones, but no significant leukocyte esterase nitrites, making the diagnosis of UTI less likely (Table 4). Blood cultures were also collected and were positive for a gram-negative rod, which then prompted the initiation of early empiric therapy with cefepime. However, due to difficulty obtaining venous access, a right internal jugular catheter was inserted to facilitate treatment administration.

**Table 1:** Complete blood count WBC: white blood count, RBC: red blood count, Hgb: hemoglobin, Hct: hematocrit, MCV: mean corpuscular volume, MCH: mean corpuscular hemoglobin, MCHC: mean corpuscular hemoglobin concentration, MPV: mean platelet volume, RDW: red cell distribution width,

Results Name	Values/Results	Units	Reference Value
WBC	1.6	thousand/mm3	4.80-10.80
RBC	3.08	million/mm3	3.10-4.50
Hgb	9.4	g/dL	10.0-14.0
Hct	26.8	%	28-42
MCV	87.1	fL	77.0-110.0
MCH	30.5	pg	27.5-33.2
MCHC	35.1	gm/dL	32-36
MPV	9.4	fL	
RDW	13.5	%	11.8-15.6
Platelet	240	thousand/mm3	150-400

**Table 2:** Comprehensive metabolic panel Na: sodium, K: potassium, Cl: chloride, CO₂: carbon dioxide, BUN: blood urea nitrogen

Results Name	Values/Results	Units	Reference Value
Na	132	mmol/L	136-145
K	4.8	mmol/L	3.7-5.3
Cl	104	mmol/L	101-111
CO2	23	mmol/L	21-31
BUN	13	mg/dL	5-24
Creatinine	0.3	mg/dL	0.7-1.3
BUN/Creatinine Ratio	43.3		
Glucose	185	mg/dL	70-140
Calcium	9.2	mg/dL	8.6-10.4

**Table 3:** Liver function tests ALT: alanine transaminase, AST: aspartate aminotransferase

Results Name	Values/Results	Units	Reference Value
Total Bilirubin	0.3	mg/dL	0.2 - 1.2
Total protein	5.6	gm/dL	6.2 - 8.0
Albumin	3.1	gm/dL	3.8 - 5.4
Alkaline Phosphatase	162	U/L	0 - 95
ALT	22	U/L	0 - 60
AST	4.7	U/L	10-36

**Table 4:** Urinalysis pH: potential of hydrogen

Results Name	Values/Results	Reference Value
Color	yellow	yellow to amber
Clarity	cloudy	clear
Specific Gravity	1.027	1.003-1.030
pH	5.5	4.6-8.0
Protein	neg	neg
Glucose	neg	neg
Ketones	trace	neg
Bilirubin	neg	neg
Nitrite	neg	neg
Leukocyte Esterase	neg	neg

During the patient’s clinical workup, a head computed tomography (CT) was performed, revealing subdural effusion without significant hypodensities or midline shift. Additionally, brain magnetic resonance imaging (MRI) showed cerebritis with characteristic findings. Following appropriate imaging, a meningitis/encephalitis panel was performed on CSF obtained via lumbar puncture, which returned positive for Hia. CSF analysis revealed significantly elevated protein levels (229 mg/dL), WBC count > 8,000 cells/µL with neutrophilic predominance (84%), and a cloudy appearance. The patient was started on vancomycin, ampicillin, and cefepime for broad-spectrum coverage of bacterial meningitis. On day 3, therapy was narrowed to ceftriaxone following pathogen confirmation.

During his hospitalization, the patient developed seizure activity characterized by tonic-clonic movements, which was managed with Ativan and later transitioned to maintenance therapy with levetiracetam (Keppra). Additionally, the central venous catheter (CVC) placed for antibiotic administration resulted in a secondary complication - a right jugular vein deep vein thrombosis (DVT). This was confirmed by ultrasound and treated with low-molecular-weight heparin (Lovenox).

By day 7, a repeat lumbar puncture showed significant improvement in CSF values, with the normalization of WBC count and protein levels. Repeat cultures suggested the removal of the infection, and the meningitis/encephalitis panel was negative. The patient continued ceftriaxone therapy until day 14 to ensure complete resolution of bacterial meningitis.

The patient tolerated therapy well, with a gradual resolution of symptoms. By discharge, the fontanelle was soft and flat, the pupils were reactive and symmetric, and tone and movement in all extremities were normal. Neurology recommended continuing Keppra as a precautionary measure, with plans for reassessment in one year.

Follow-up imaging, including brain MRI, revealed stabilization of cerebritis with no progression or new findings. At his two-year follow-up, the child demonstrated normal development and remained seizure-free, allowing Keppra to be discontinued. The patient remains under routine follow-up and is doing well without any evidence of neurologic deficits.

## Discussion

*H. influenzae* is a small, facultatively anaerobic, pleomorphic, capnophilic, gram-negative coccobacillus belonging to the family Pasteurellaceae [2]. It is an obligate human pathogen that primarily colonizes the nasopharynx and, to a lesser extent, the genital tract and conjunctivae [1]. As previously discussed, *H. influenzae *can be broadly classified into the encapsulated (serotypes a-f) and NTHi forms [2]. The most significant encapsulated serotype is Hib, whose virulence is primarily attributed to its PRP capsule. This subtype accounts for 95% of invasive *H. influenzae *diseases in children and over 50% in adults. In contrast, NTHi is a less frequent cause of infection [2].

Before the introduction of the Hib protein conjugate vaccine, Hib was the leading cause of bacterial meningitis in young children. In the pre-vaccine era, the incidence of Hib meningitis in U.S. children aged 0 to 4 years was approximately 54 per 100,000 annually. However, widespread immunization efforts have drastically reduced invasive Hib disease by over 90% in high-income countries [1]. However, lower-income regions initially faced delays in adopting the vaccine. Global efforts have expanded immunization, achieving nearly universal coverage by 2020, except in certain regions such as China and Russia.

Despite Hib's decline, invasive infections caused by other *H. influenzae* serotypes, particularly Hia and NTHi, have increased. Between 2002 and 2015, the U.S. saw annual increases of 13% and 3% in Hia and NTHi infections, respectively. Current vaccines target Hib but do not cover other serotypes [1]. These recent trends highlight the need for research into vaccines for non-Hib serotypes as their clinical significance grows in today's world.

Although *H. influenzae* can affect individuals of all ages, infants and adults over 65 are at particularly high risk. There also appears to be increased susceptibility among certain racial groups, including the American Indian and Alaska Native groups. Risk is further elevated in various immunocompromised states, including sickle cell disease, human immunodeficiency virus (HIV), asplenia, complement deficiency, and in individuals receiving chemotherapy or radiotherapy [2].

Unlike other pathogens, *H. influenzae*’s treatment strategies vary depending on the severity of infection, requiring tailored approaches to address different clinical presentations. Broad-based coverage for treating *H. influenzae* includes beta-lactams, fluoroquinolones, macrolides, and tetracyclines. In cases of localized, non-severe symptoms, such as sinusitis or otitis media, the primary empiric treatment of choice is oral amoxicillin-clavulanate, with oral second or third-generation cephalosporins (cefuroxime, cefdinir, cefixime, or cefpodoxime) being suitable alternatives. In contrast, systemic infections, such as meningitis, epiglottitis, or bacteremia, must be treated with intravenous third-generation cephalosporins (ceftriaxone) [3].

Almost always, meningitis is empirically treated before confirmation of the specific pathogen responsible for the infection. Currently, empiric treatment standards involve ceftriaxone and vancomycin, with ampicillin added if *Listeria* is suspected. Ampicillin is generally not the first-line treatment for severe *H. influenzae* infections due to its limited efficacy against strains with high beta-lactamase production. This resistance makes ampicillin less effective unless beta-lactamase testing confirms a negative result [3].

Empiric treatment is not specific to meningitis cases but can also be used for non-identified bacteremia. As mentioned in this case, the patient received empiric cefepime due to unidentified gram-negative bacteremia. After further lab results identified meningitis secondary to bacterial infection, treatment was adjusted to include vancomycin and ampicillin alongside cefepime to ensure broad coverage. On the third day of treatment, therapy was tailored to ceftriaxone following confirmation of *H. influenzae*. Dexamethasone was administered to manage meningitis and neuronal injury, helping prevent further complications through its anti-inflammatory effects. Its use requires precise clinical judgment and careful timing. Evidence also indicates that the combination of dexamethasone and antibiotics reduces neurological sequelae [4].

Both the patient’s diagnosis and course of treatment were straightforward and provided a safe and effective approach to minimizing the risk of long-term complications. However, even the most routine treatments for severe *H. influenzae* infection are not without risk, particularly the use of CVCs, which are commonly used in pediatric and intensive care units (ICU) for the infusion of medications, blood products, and fluids. Although generally safe, CVC use must be carefully balanced against its risks, requiring close monitoring and clinical judgment. In children, peripheral venous access may be limited, making CVCs especially critical in emergent situations where delayed IV access can lead to complications or even death. However, CVCs themselves can cause complications, and this patient, in particular, dealt with DVT secondary to CVC usage, which was presumed to be iatrogenic. This further complicated and delayed treatment for the patient, underscoring the need for vigilant monitoring, especially in urgent situations.

Generally, the development of DVT is fundamentally explained by Virchow’s triad, which identifies three primary risk factors that favor clot formation: venous stasis, endothelial injury, and a hypercoagulable state [5]. In this case, the CVC was the suspected cause of DVT in the patient. As previously mentioned, vascular injury increases the risk of DVT by promoting the release of procoagulants, enhancing coagulant production by the liver, and reducing tissue plasminogen activator (tPA) production. The incidence of CADVT is higher in pediatric populations, affecting 2-26% of cases. Among various pediatric risk factors, research suggests that intracranial space-occupying lesions, bleeding, and infections or inflammatory diseases significantly increase CADVT events. Additional factors, such as the length of ICU stay, duration of CVC use, catheter type, and improper placement, also elevate the risk of CADVT. In this case, the patient - a 10-week-old with Hib meningitis and complications such as tonic-clonic seizures - was at exceptionally high risk for DVT due to these factors [6].

Seizures can arise from the *H. influenzae* meningitis disease process and represent a significant complication in its management. Seizures are transient episodes of abnormal, excessive, or synchronous neuronal activity in the brain that can result in a variety of clinical manifestations. They can be a symptom of an underlying neurological or systemic disorder or occur as a primary condition, like epilepsy.

Seizures are classified into three main types: focal (partial) seizures, generalized seizures, and idiopathic seizures. Focal seizures are further divided into focal aware seizures and focal impaired awareness seizures. Generalized seizures include tonic-clonic, absence, myoclonic, atonic, and isolated tonic or clonic seizures.

Seizures result from disruptions in brain function due to an imbalance in the homeostatic neuronal network, occurring in the setting of either excessive excitation (glutamate activity) or impaired inhibition (gamma-aminobutyric acid dysfunction) of neurotransmission. This imbalance leads to involuntary movements, abnormal sensory experiences, and erratic emotions or behavior [7]. Seizures are highly associated with epilepsy, high fever (febrile seizures), head injuries, infections (e.g., malaria, meningitis, and gastrointestinal illness), as well as metabolic, neurodevelopmental, and cardiovascular conditions, and complications associated with birth [8]. Furthermore, seizures are more prevalent in the pediatric population, with significant associations with the risk factors listed above, further highlighting the need for careful management [9].

Seizures secondary to bacterial meningitis are multifactorial and result from disruptions in neurotransmission. Bacterial meningitis induces significant inflammation as the host immune system responds to bacterial products, leading to increased intracranial pressure. Inflammation, bacterial products, and elevated intracranial pressure disrupt physiologic neurotransmission ultimately leading to seizures [9].

As previously noted, up to 50% of survivors develop neurological complications, with outcomes highly dependent on the patient's age and the infecting organism [1]. Neurological complications of meningitis can include hearing loss, vision impairment, seizures, hydrocephalus, behavioral and emotional disorders (e.g., attention-deficit/hyperactivity disorder, mood disorders, social difficulties), cognitive impairments, and motor disabilities (e.g., cerebral palsy). These complications largely result from inflammation caused by the host’s immune response to the infection. Hearing loss is the most common sequela, resulting from inflammation in the cochlea and cranial nerve VIII, as well as bacterial products disrupting the labyrinthine blood barrier [10]. Managing these complications requires ongoing follow-up with a general practitioner, pediatrician, and neurologist. While no standardized protocol currently exists, further research is needed to determine the best strategies for tracking and minimizing the negative sequelae of the disease.

## Conclusions

In conclusion, Hia meningitis remains a rare but serious condition with significant potential for complications, particularly in the pediatric population. This case underscores the importance of timely diagnosis and tailored treatment strategies. Despite the reduction in Hib meningitis due to widespread vaccination, Hia and other non-Hib serotypes are increasingly contributing to invasive infections across the world. The patient's complete recovery, despite complications such as cerebritis, seizures, and CADVT, highlights the importance of early intervention, appropriate antimicrobial therapy, and multidisciplinary care. Continuous monitoring and long-term follow-up are essential to minimize sequelae, particularly neurological deficits, and to ensure optimal clinical outcomes.
